# Women’s experiences of their pregnancy and postpartum body image: a systematic review and meta-synthesis

**DOI:** 10.1186/1471-2393-14-330

**Published:** 2014-09-23

**Authors:** Emma L Hodgkinson, Debbie M Smith, Anja Wittkowski

**Affiliations:** School of Psychological Sciences, Second Floor Zochonis Building, Brunswick Street, Manchester, M13 9PL UK; School of Psychological Sciences, Room 1.17, Coupland 1 Building, Oxford Road, Manchester, M13 9PL UK; Manchester Mental Health and Social Care NHS Trust, Manchester, UK

**Keywords:** Body image, Thematic synthesis, Meta-synthesis, Mothers, Postpartum, Pregnancy

## Abstract

**Background:**

Pregnancy-related physical changes can have a significant impact on a woman’s body image. There is no synthesis of existing literature to describe the intricacies of women’s experiences of their body, and relevant clinical implications.

**Methods:**

Four electronic databases were searched in February 2014 using predefined search terms. English-language, qualitative studies published between January 1992 and December 2013 exploring pregnancy and postpartum body image were included. Following quality appraisal, 17 papers were synthesised using the interpretive thematic synthesis approach within a social constructionist framework.

**Results:**

Three themes were highlighted: “Public Event: ‘Fatness’ vs. Pregnancy”, “Control: Nature vs. Self”, and “Role: Woman vs. Mother”. Women perceived the pregnant body to be out of their control and as transgressing the socially constructed ideal, against which they tried to protect their body image satisfaction. Women perceived the physical manifestation of the mothering role as incongruent to their other roles as a wife or partner, or working woman. Body dissatisfaction dominated the postpartum period.

**Conclusions:**

Women’s perception of their pregnancy body image is varied and depends on the strategies they use to protect against social constructions of female beauty. Women have unrealistic expectations for their postpartum body, highlighting this as an area where women need better support. Attending to women’s narratives about their pregnant body may identify at-risk women and provide an opportunity for health professionals to provide support to either address or accept body image dissatisfaction. Clinical communication training may enable health professionals to explore body image concerns with women and guide them in identifying ways of accepting or reducing any dissatisfaction.

## Background

A woman’s body image is a psychological representation of her body comprising her attitudes and self-perceptions of her appearance [1], developing from biological, psychological and social influences [1–3]. It can be significantly affected by the rapid and extensive physical changes during pregnancy and postpartum [4, 5], signifying a transition to motherhood parallel to the psychological assimilation of this role. Pregnancy is a complicated period for women in that it is often the first time weight gain is expected and accepted. A social constructionist approach to body image explains the ideal body as a socially constructed phenomenon when physical characteristics are imbued with moral judgements [6]. A discrepancy between women’s body image and this constructed ideal can cause body dissatisfaction [7], possibly leading to significant psychological distress [8].

There is a lack of research exploring the specific impact of pregnancy on body image. Some traditionally used body image assessments have been shown to be inaccurate during pregnancy [9], making body image in pregnancy more difficult to assess, both clinically and in research. Modified measures, however, have been used successfully, and existing studies have identified that women’s body satisfaction is higher during pregnancy [10]. A review of the literature indicates that body dissatisfaction in pregnancy is associated with a number of factors including low mood, importance of body image, perceived socio-cultural pressure, intention to breast feed and eating restraint [11]. Although this research allows the objective analysis and quantification of body image in both a cross-sectional and longitudinal approach, it does not capture the idiosyncrasies of women’s experiences that a qualitative approach does. To date, no reviews have been published exploring the psychological impact of pregnancy on body image as identified in the qualitative literature, and no attempt has been made to collate the existing literature into a usable basis for clinically relevant guidance. The aim of this metasynthesis was to review and explore studies of women’s perceptions of their body image during pregnancy and postpartum, and to highlight the clinical implications for health professionals working in obstetrics. *Primary research question*: What are women’s experiences of body image in pregnancy and the postpartum phase? *Secondary research question*: What are the clinical implications of women’s perceptions of their pregnant body and how can obstetric services and other relevant health settings support women?

## Methods

### Search strategy and selection criteria

Studies were limited to those published in English between January 1992 and December 2013 to include recent changes in recommendations for weight management in pregnancy. Included studies were qualitative (pure or mixed methods), utilising a sample of women over 16 years of age without a specified psychopathology. For the purposes of this metasynthesis, the postpartum phase will be considered as up to two years following childbirth as defined by the National Institute for Health and Care Excellence [NICE] guidelines [12] for weight management.

**Figure 1:**
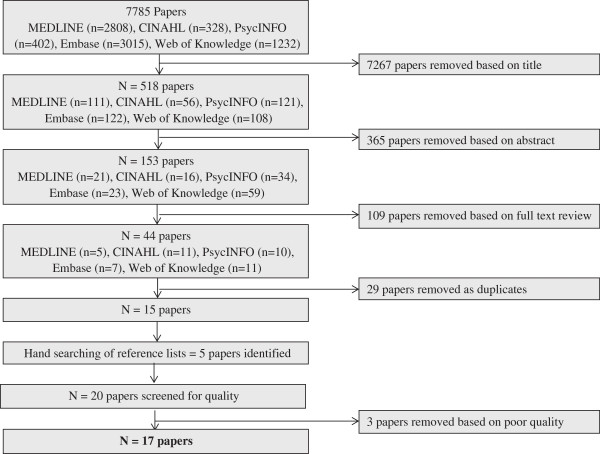
**Flow diagram showing the search method and exclusion process.**

Online databases PsycINFO, MEDLINE, Web of Knowledge, and Embase were systematically searched by author EH. Keywords were truncated, and multiple synonyms of search terms were used as appropriate. Reference lists of identified papers were hand-searched, and reference and citation functions were used where available. Broad search terms were selected to capture a wide range of papers and followed the PICO search tool (namely Population and Outcome): [pregnan* OR postnatal OR postpartum OR prenatal OR antenatal OR *gravida* OR *parous] AND [appearance OR body image OR body dissatisfaction]. The search was conducted in February 2014 according to the flow diagram in Figure 1. Fifteen studies were yielded by the search of the databases described above, with a further five papers identified from hand-searching the reference lists of the included papers. The researchers adhered to PRISMA guidelines for systematic reviews and metasyntheses. Ethical approval was not required for this systematic review.

### Quality appraisal

Two of the authors (EH and DS) independently screened the 20 studies, using Walsh and Downe’s quality appraisal tool [13] and the Critical Appraisal Skills Programme (CASP) checklist [14] to assess different aspects of methodological and interpretive rigour (e.g., for appropriate research design, clear statement of aims, rigorous analysis, ethical considerations, adequate recruitment and data collection, and evidence of reflexivity). Papers were graded on a range of items as follows: A = no significant flaws, B = minor flaws not affecting credibility or validity, C = some flaws likely to affect credibility or validity, and D = significant flaws affecting credibility or validity. There was disagreement of one grade difference between the two reviewers’ ratings for nine papers, which were discussed to yield a single rating, finalised by all authors (see Table 1). After a review by the three authors to ensure scientific value was not lost [15], it was decided to exclude three papers assigned a final rating of ‘D’ [16–18]. Details of the final 17 studies are summarised in Table 1.

**Table 1:** **Characteristics of the studies included in the metasynthesis**

Author(s)	Focus	Sampling; Context	Sample size	Ethnicity; SES	Age	Marital status	Parity	Data collection; Data analysis	Theoretical framework	Quality rating
Nash (2012) Australia [22]	Examine early pregnancy embodiment and BI	Self-selection in response to advert; City	N = 38	White; Middle class	21-40	NS	NS	SSI at 10 week intervals from 10/20 weeks gestation to post birth; Situational analysis	Feminism	B
Harper & Rail (2011) Canada [35]	Young women’s discursive construction of the pregnant body in the context of obesity	Snowball sampling, prenatal classes; City	N = 15	NS; NS	18-28	NS	n = 13 primi- & n = 2 multiparas	SSI during pregnancy; Thematic and discourse analysis	Feminist post-structuralism	B
Ogle et al. (2011) US [37]	The meaning and implications of the pp body for married couples	Snowball sampling; Two towns, clinic and community	N = 14 couples	24 Caucasian, 1 Asian, 2 Hispanic; Middle class	22-39	Married	N = 14 primiparas	Separate SSI 28 – 36 weeks gestation, and 2-6 weeks pp; Hermaneutic approach	Interactionist/dramaturgical	B
Carter (2010) US [24]	Concept of control body/self in pregnancy and childbirth	Theoretical and snowball sampling; Birth centres	N = 18	3 Hispanic, 1 African American, 10 White; n = 13 upper/middle, n = 5 lower middle/ working class	NS	NS	n = 15 primi- (n = 1 with adopted child) and n = 3 multiparas	SSI at 6-18 months pp; Narrative analysis	Social constructionism	B
Clark et al. (2009) Australia [25]	Women’s experience of the body in pregnancy and pp	Social network snowball sampling; Two cities	N = 20	n = 19 Australian, n = 1 British; NS	21–42	n = 16 married, n = 4 cohabiting;	n = 18 primi- & n = 2 multiparas; n = 10 pregnant, n = 10 pp	SSI – gp1 = 30–38 weeks gestation; gp2 = 5-12 weeks; Phenomenology, thematic content analysis	Phenomenology	A
Chang et al. (2006) Taiwan [26]	Body image of Taiwanese women in the third trimester	Purposeful sampling; Prenatal examinations	N = 18	Taiwanese; NS	21-45	n = 17 married, n = 1 engaged	n = 15 primiparas & n = 3 multiparas	SSI in 3rd trimester; Phenomenology	Phenomenology	A
Johnson et al. (2004) UK [27]	The meaning of body change for first time mothers to be	Convenience sampling; Email to colleagues	N = 6	1 British Asian, 5 White; All working for a university	26-34	Married	N = 6 primiparas	SSI at 33-39 weeks gestation; Phenomenology & Foucaldian discourse analysis	Phenomenology & Foucaldian discourse analysis	B
Seibold (2004) Australia [28]	The experiences of young pregnant women, maternal embodiment and identity construction	Convenience sample; prenatal classes, city hospital	N = 5	NS; NS	17-23	n = 4 single, n = 1 unmarried partner	Parity not stated; n = 5 pregnancies unplanned	SSI at 24-26 weeks gestation, 6-8 weeks and 6 months pp (telephone); diaries in 3rd trimester; Organising themes	Feminism	B
Earle (2003) UK [29]	Physical appearance concerns during pregnancy	NS; 12 antenatal clinics	N = 19	n = 1 Asian, n = 18 White; Range	16-30	NS	N = 19 primiparas	Unstructured interviews at 6–14, 34–39 weeks gestation; Grounded theory	NS	B
Upton & Han (2003) US [23]	The perceived boundary between self and body after pregnancy	Snowball sampling; Urban community	N = 60 couples	NS; Middle class	26-34	Married	NS	n = 52 Ethnographic interview, n = 8 interview & observation; Ethnography	Phenomenology	B
Bailey (2001) UK [38]	Gender in pregnancy and postpartum	Snowball sampling; Antenatal classes	N = 30	White; Middle class	25-38	NS	N = 30 primiparas	SSI at 3rd trimester and 3-6 months pp; Content analysis	Feminism	B
Bondas & Eriksson (2001) Finland [30]	The lived experiences of pregnancy	Purposeful sampling; NS	N = 40	NS; NS	NS	NS	N = 40 primi- & multiparas	SSI at 36th week gestation, 3 weeks, 3 months and 2 years pp; Phenomenology	Phenomenology	A
Schmied & Lupton (2001) UK [31]	How body image gives meaning to the embodiment of pregnancy	Convenience sampling; city hospital	N = 25 couples	1 Brazilian, 1 German, 23 British; n = 15 white collar occupations, n = 12 educated to degree level	23-35	Cohabiting	N = 25 primiparas; n = 8 unplanned, n = 17 planned	SSI in pregnancy, 2-10 days, 4-8 weeks, 12-14 weeks, and 5-6 months pp; Identifying themes and patterns	Post-structuralism	B
Devine et al. (2000) US [32]	How women experience weight change in pregnancy and pp	Flyers in nutrition clinics; City	N = 36	n = 1 Asian, n = 35 American; n = 24 employed, n = 8 university & n = 2 high school students	18-40	n = 33 married, n = 3 NS	n = 27 primiparas & n = 9 multiparas	SSI mid-pregnancy, 6 weeks, 6 (telephone) & 12 months pp; Constant comparative method	Interpretivist	A
Bailey (1999) UK [33]	Identity in the transition to motherhood	Snowball sampling; Antenatal classes	N = 30	White; Middle class	25-38	n = 29 cohabiting, n = 1 single	N = 30 primiparas; n = 6 unplanned, n = 24 planned	SSI at 3rd trimester; Discourse analysis	NS	B
Fox & Yamaguchi (1997) UK [34]	BI changes experienced by normal and overweight women in pregnancy	Convenience sampling; Four hospitals	N = 76 (42 - BMI 20–24, 34 - BMI 25–39)	57 White, 16 Black, 3 Indian Asian; n = 23 professional, n = 23 skilled, n = 12 partially/ unskilled, n = 18 unemployed	18-27	NS	N = 76 primiparas	Mixed methods questionnaires completed at least 30 weeks gestation; Identification of themes	NS	B
Wiles (1994) UK [36]	The impact of pregnancy on women’s feelings about weight	Identification by midwives, postal; Two hospitals	N = 37 weighing >90 kg by 30th week	White; NS	21-30	n = 30 cohabiting, n = 6 living with parents, n = 1 single	NS	SSI and free text questionnaire at 30-40 weeks gestation; NS	Feminism	C

(primaparous = in their first pregnancy, or if postpartum, have one baby; multiparous = not first pregnancy or have more than one baby; SSI – semi-structured interviews; pp - postpartum; SES – socioeconomic status; NS – not stated). Studies listed in chronological order - numbering of paper in column one corresponds to number in reference list.

### Synthesis

**Figure 2:**
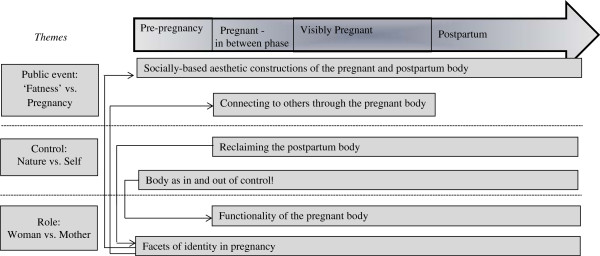
**Representation of the synthesised themes and higher level interpretive themes, and their presence during pregnancy and the postpartum phase.** Arrows indicate links between the themes highlighting that the content and implications of each theme are interconnected and thus cannot be taken in isolation. ‘Pre-pregnancy’ – the time before becoming pregnancy; ‘Inbetween’ – the initial phase in the first trimester before the woman’s expanding stomach is clearly visible; ‘Postpartum’ – up to two years after childbirth.

An interpretive thematic synthesis approach was used to synthesise the original content of the studies and generate a higher level of understanding through interpretive themes [19]. The thematic synthesis was conducted in three stages [19]. Two authors independently coded the studies to capture the themes within the original studies relating to women’s experiences of their physical body during pregnancy and postpartum. The initial set of themes was discussed and explored in terms of frequency of endorsement, and similarities and differences, and grouped into a hierarchical structure (Figure 2). A secondary level of descriptive themes comprising themes with similarities in content and meaning was generated, reviewed and discussed within the research team. A tertiary level of interpretive themes was developed through further discussions. As retaining the context of the original data is essential when extracting the true meaning from the studies [20], validity of the themes was ensured by re-reading the studies and drawing comparisons.

The authors were mindful of their own experiences and views of pregnancy and body image throughout the process of the synthesis [21]. All authors were female and of childbearing age, two of whom had no experience of pregnancy and motherhood. Two authors had expertise in clinical psychology, with the third having a health psychology background; thus, both physical and mental health angles were explored.

## Results

### Included studies

Characteristics for the 17 papers [22–38] can be seen in Table 1, based on a total of 487 women aged between 16 and 45 years old. Two of the papers resulted from one recruited sample [33, 38], with the remaining 15 papers resulting from 15 separate samples. Although two papers included data from heterosexual married couples [23, 37], themes pertaining to the women only were reported because interviews were conducted with husband and wife separately. All other interviews were conducted with women only. All studies included in this review were conducted in developed countries (UK n = 7, Australia =3, USA n = 4, Taiwan n = 1, Finland n = 1, Canada n = 1), meaning the women sampled in these studies were likely to have experienced their pregnancies within a context of pregnancy-related healthcare involving assessments of psychological and physical wellbeing, one of which may have been weight management, within mixed private and free healthcare systems. Within the quality appraisal, only one study [36] was assigned a ‘C’ rating, indicating it may be necessary to interpret these results with greater caution.

### Findings

Following the metasynthesis process outlined above, women’s experience of their physical body could be defined in three ways: *“Public Event: ‘Fatness’ vs. Pregnancy”, “Control: Nature vs. Self”,* and *“Role: Woman vs. Mother”*. Supporting quotes are presented in Table 2.

**Table 2:** **Key quotes from the studies to illustrate the synthesised themes**

Description	Quote
*Public Event: ‘Fatness’ vs. Pregnancy*	
Connecting to others through the pregnant body	“There is just enormous connection between women who’ve had children… it’s like becoming part of a club …” [31]
	“It was as if suddenly the whole world had access to my body…” [23]
Socially-based aesthetic constructions of the pregnant body	“Having the big tummy during pregnancy was fine, I enjoyed that, because it meant I was pregnant and everyone could see that. But now, if I’m not with my baby then people have no idea why I’m bigger” [25]
	“I haven’t put a lot of weight on. It feels more than it actually is. I’ve been lucky. It is literally all baby. Solid.” [29]
	“People don’t look at you so disgustingly when your’re pregnant when you’re wearing something like shorts as they do when you’re vastly overweight” [36]
	“I hated to be pregnant, ugh. I thought it was disgusting” [30]
	“I love my new shape. I have always been quite small, so I prefer having some shape and curves” [34]
*Control: Nature vs. Self*	
Boundaries between the self, the body, and the baby	“It’s a bit like the invasion of the body snatchers” [38]
	“I’m becoming aware that within, it’s developing its own personality. Its becoming, I suppose, less and less dependent on me or less and less a part of me and more an individual” [31]
	“constant companion” [31]
Body as in and out of control	“I guess that was one of the hardest things…just the whole sense of losing control over your body and nothing you can really do is going to stop this process from happening…” [24]
Reclaiming the postpartum body	“I would have liked to have known that I wasn’t going to lose weight again quickly after having her… I just didn’t know these things…” [25]
	“It was like I had double the work…I was back to square one, but worse” [23]
	“You’re always trying to get it back, but never really can have it back” [23]
	“Some women just can’t get their old shape back at all, and I’m bound and determined not to be one of them” [37]
*Role: Woman vs. Mother*	
Facets of identity in pregnancy	“not changed; just probably deepened. Deepened in the sense that I’m probably aware of myself in a very different way, which is valuable” [33]
	“I actually wanted to cover up, I wanted to present myself as not being pregnant…I’d this sort of professional image and I’d not let the pregnancy get in the way” [38]
	“I’m still me, really, not just a pregnancy” [33]
	“So almost everything I do is really about being pregnant in some way.” [38]
	“… there were lots of ways that I felt very ambiguous about my sexuality and my sort of like being a woman, and I suppose thinking for the two years before that I couldn’t have children had played into all those feelings that I’d had…not being a proper girl, and not being a proper woman. So [the pregnancy] confirmed – I mean, it did reverse that”. [38]
	“They [breasts] don’t add to you being a woman anymore, they’re just practical…I suppose they’ve lost – lost something sexually maybe” [38]
	“Husbands always expect their wives to be pretty. But, I can only wear maternity clothes and cannot dress up because my body shape has changed for pregnancy.” [26]
Functionality of the pregnant body	“I felt like my body was preparing to look after a child, it was making a child! I was thinking ‘there is a human being developing here, and my body is doing it!’ It is phenomenal! There is nowhere else that can actually incubate and grow a human being, that’s what your body does. I was nourishing it, and it was just amazing…its mind blowing.” [25]

#### Public event: ‘fatness’ vs. pregnancy

This theme, comprising two subthemes, conceptualised the way women’s perceptions of their bodily changes were influenced by their perceptions of socially constructed ideals, and the active involvement of the public in women’s pregnancies.

### Socially-based aesthetic constructions of the pregnant body

The selected studies described women as perceiving socially constructed ideals to be of thinness, shapely breasts and unmarked skin [22, 25], and of controlling weight through diet and exercise [29]. Women acknowledged changes in their body as an inevitable part of pregnancy [29], but clearly differentiated between the social unacceptability of being fat versus the social acceptability of being pregnant [34], with the pregnant body transgressing these socially constructed ideals. The studies described women’s pregnant body image as protected from this transgression, because the women legitimised it through perceiving themselves as excused from adhering to the ideals or by reflecting on the functional and mothering identities of their body [34]. Those who had been overweight before their pregnancy were protected by these strategies, with women viewing their pregnancy as excusing them from unpleasant comments or feeling uncomfortable in activities exposing their body, for example, swimming [37]. Pregnancy-related weight was described as weight attributable to their developing fetus, whereas ‘fatness’ was viewed as all other weight gained (e.g., arms or faces [29]). The latter was perceived as gaining unnecessary weight, which was explained by the studies as harder for women to legitimise, resulting in body dissatisfaction [25].

Difficulties were reported in negotiating the first trimester of pregnancy when the waist thickens but the pregnancy is not yet visible [22]. Women perceived their expanding stomach as the ultimate confirmation of their pregnancy and during this “in-between phase” [22], feeling that no one would recognise their excuse for having a larger body. This perception was mirrored in the postpartum period, when women’s constructions of their postpartum body indicated that once the baby was born, they no longer perceived any excuse not to adhere to their perceived socially constructed body ideal [23].

Despite the women feeling they had an excuse during pregnancy, and the distinction between fatness and pregnancy, studies demonstrated women as having a continued awareness of the socially constructed ideal. An increased breast size moved women towards the socially constructed ideal, increasing body image satisfaction [26], whereas changes such as acne or stretch marks moved them away from this ideal [26, 30]. Three studies acknowledged a representation of a new construction of women’s bodies that did not necessarily conform to the perceived social ideal but was still beautiful through its curves and rounded shape [29, 34, 35]. There were only two instances where explicit dislike of the expanding stomach was expressed, acknowledging this may not be a socially acceptable comment [29, 30].

### Connecting to others through the pregnant body

As well as influencing women’s perception of their own bodies in comparison to their perceived social norm, members of society were found to be actively involving themselves in women’s pregnancies. Women perceived their bodies as public property during pregnancy, with family, friends and strangers touching their stomach or making personal comments about their appearance or behaviours [27]. Although the dissolution of this aspect of social boundaries was reported as invasive, enjoyment was expressed due to the closeness and connection they felt with other pregnant women [33]. Juxtaposed against this perceived compression of social boundaries was the reported extension of physical boundaries, with members of society described as offering women greater physical space than their body required due to a perceived vulnerability of the pregnant body [38].

#### Control: nature vs. self

This theme conceptualised the way the women experienced pregnancy as striving for control against nature, with nature being the driving force behind the growth of the fetus and the pregnancy-related bodily changes. Two subthemes highlight women’s perception of control over their body and foetus, and their efforts to exert control over their body’s weight and behaviour both during pregnancy and in the postpartum period.

### Body as in and out of control

The studies framed women’s bodies as separate and autonomous agents undertaking the pregnancy-related changes of their own accord [31], sometimes to the point that women considered their body had become a stranger to them [25]. The theme of women’s bodies being perceived as out of control during pregnancy dominated. Four studies reported women as focusing on their weight gain and becoming anxious if they perceived they were not meeting others’ weight gain expectations, either because they felt they were gaining too much or too little [22, 26, 36, 37]. Comments from health professionals were reported to reinforce the belief that women should be in control of their bodies and their weight during pregnancy [22], particularly if women presented with problems including high blood pressure, perineal tearing [24] or excessive gestational weight gain [22]. In these 17 studies, when women viewed themselves as being unable to be in control, they reported to experience distress [24]. However, this distress was mediated if they perceived their body to be accommodating of their own needs; for example, when they needed a break from contractions during labour [24].

Another important aspect emerging from the studies was a perceived loss of control over the body, having to share it with the fetus. Women reported different relationships with their fetus. Two distinct groups were identified in terms of women’s attitudes towards the presence of the baby in their body: sharing and invasion. Some women expressed comfort in sharing their body with their fetus [31], whereas others reported perceiving their fetus as invasive [31]. These groups seemed mutually exclusive because women did not report holding onto both beliefs at once. Furthermore, some women found it difficult to identify the fetus as a separate being, even late in pregnancy.

### Reclaiming the postpartum body

Studies identified that even during pregnancy, women reported that society expected them to reclaim control of their body following the birth of their baby, and described this as a distressing and fearful prospect [25]. The postpartum body was portrayed as a project to be actively worked on and controlled to get back to normal [25], with many women perceiving this to be a bigger goal postpartum than before pregnancy [23]. This was noted as true both for multiparous women, who reported they had already reclaimed their body from previous pregnancies, and for primaparous women. One woman commented that she perceived the people around her as expecting them to exert control to such a degree that their postpartum body was slimmer than their pre-pregnancy body [23]. The expectations held by many women for their postpartum body were high, and were often recognised as unrealistic. However, only two studies reported that women believed the mothering role caused them to reprioritise their body image below their baby [37, 38].

#### Role: woman vs. mother

According to this theme, pregnancy was described as a period in which women negotiated their existing roles and identities, integrating their new role as a mother. Two subthemes underpin this theme.

### Facets of identity

According to the studies, pregnancy caused women to become more aware of the different roles and facets of their self rather than lose their core self. Two specifically commented on this in terms of their femininity and womanhood. Pregnancy was described as a confirmation of womanhood, particularly for those who had experienced gynaecological or conception difficulties [33, 38].

The transition into the mothering role marked by pregnancy-related changes was noted to be perceived by women as incompatible with their other roles, such as their core self, being a wife or sexually attractive woman, or being a working woman. Comments from husbands were reported to influence whether women believed their physical manifestation as a mother no longer enabled them to be attractive to their husbands, despite commenting on the way some bodily changes moved them towards the socially constructed body ideal. This was further reflected in the way studies identified some women as considering that their postpartum body conferred beauty associated with a mothering identity rather than the socially accepted ideal for sexual attractiveness [33]. Perceived support was associated with increased satisfaction with appearance with respect to women’s relationship with their partner, whereas those who felt criticised were reported to be less secure [26]. The studies also highlighted that men influenced women’s perceptions of their working identity. Working women were described as actively adopting a “male identity” to conform to their perception of the male-dominant workplace in order to progress in their career. Pregnancy was expressed as actively renouncing this identity and re-allocating the values from work, such as responsibility, stability and interest, into motherhood [33]. The studies highlighted that this was considered undesirable by many women due to the perceived detriment to their career, resulting in women covering up their pregnant body to preserve their working relationships and work persona [38]. Indeed, four studies reported women did not seem to wish to assert their pregnant identity through maternity clothing, but preferred to use clothing to express their individuality [23, 27, 29, 30].

### Functionality of the pregnant body

Women were seen to adopt their mothering role through acknowledging the newfound functionality in their bodies. They seemed to consider their expanding stomach as a proxy for their baby’s health and growth, and were noted to be anxious about their baby’s wellbeing if they perceived their own stomach was small in comparison to other women’s [30]. A number of studies described women’s awe and fascination in the functional adaptations their body made during pregnancy [34, 27, 29].

## Discussion

### Main findings

This exploration of women’s experiences of their pregnant body suggests that body image is a product of socially constructed ideals. Women perceived pregnancy as a transgression of these ideals, but protected their body image by delineating between ‘fatness’ and pregnancy, feeling excused from conforming to the socially constructed ideal. These reasons were not considered as prominent postpartum or in the first trimester, therefore dissatisfaction and striving to reclaim the postpartum body dominated, with studies highlighting that women strongly believed that “pregnancy is socially acceptable but being fat is not” [34]. Furthermore, pregnant bodies were noted to be perceived by women as beyond their control, whilst they received messages they should be in control. The studies identified that women described the pregnancy-related changes as causing a re-negotiation of their identity, moving them away from identities of being a sexually attractive woman, and towards a mothering identity.

### Strengths and limitations

One strength of this meta-synthesis is that it provides a relevant interpretation of a range of women’s views, with considerations for discussing weight management during pregnancy. It is vital that women’s views are incorporated in the development of maternal obesity services [39, 40] in order to improve their acceptability and effectiveness. This meta-synthesis bridges the gap between theory and clinical practice, providing a basis for future clinical guidelines.

However, there are a number of potential limitations to be considered. The studies were mainly published in the USA, UK, and Australia. It can therefore not be assumed the findings will apply to countries where healthcare resources and attitudes to body image differ. In addition, a number of the studies identified their sample as being middle class, with only a small number of studies recruiting women from other socioeconomic backgrounds (see Table 1). Some of the findings may therefore not represent the views of women from other countries and across a full range of socioeconomic backgrounds.

Although methodological quality was taken into account through appraisal, there was notable variation in the studies. Studies consistently failed to adequately highlight the role of the researcher(s) and their impact on the interpretation of the data. Equally, sufficient demographic information was sometimes unavailable, making it harder to interpret findings within their original socio-demographic context.

Although the studies recruited prima- and multiparous women, no differences between themes were drawn between these groups. Pregnancy-related changes are most noticeable after the first pregnancy, and failure to lose postpartum weight is a predictor of long-term obesity [41]. Consequently, it is important to understand how women of different parities respond to bodily changes as well as their attitudes towards mothering to provide support tailored to their specific needs.

### Interpretation

According to the “looking glass self” theory [42], self-image is constructed from the one reflected back by society. The findings of this meta-synthesis suggest that society has mixed views towards the pregnant body, oscillating between positivity and disgust. Women’s perceptions of their pregnant bodies may be influenced by societal stigma towards obesity, especially as negative characteristics, such as being lazy, are attributed to obese people [43, 44].

Bodies are typically constructed by society as controllable, particularly with respect to weight [42], but the pregnant women in these studies did not describe this. It has been suggested that society drives women towards achieving the socially constructed body ideal through control of bodily functions [45] to protect against fear of mortality, and that pregnancy poses an existential threat by exposing them [46]. Given that pregnancy-related changes are inevitable, there is a widening gap between the “imperfections” of the pregnant body and societal expectations. Therefore, negotiating the perceived loss of socially prescribed attributes associated with the pre-pregnancy body against actively developing a helpful mothering orientation may become more difficult. Health professionals should be aware of the societal pressures concerning striving towards the perfect body and the implications of this for women’s pregnant and postpartum body image. Pregnant women may feel pressured to over-invest in body management strategies. Thus, paying attention to narratives around seemingly less notable pregnancy-related changes such as stretch marks, flushing, or swollen ankles as well as weight gain may facilitate discussions about the women’s adjustment to her pregnancy and bodily changes. Interestingly, while many of the pregnancy-related bodily changes were considered by women to move them away from the ideal body image, weight gain was perceived as more socially acceptable. Although this protects against body dissatisfaction, it reduces the motivation to achieve moderate weight gain for those at risk of excess gestational weight gain. Despite this difficulty, health professionals are reported to feel uncomfortable about discussing weight as an aspect of body image due to lack of knowledge and fear of being considered insensitive [39, 47]. Evidence suggests it is less stigmatising to focus on a healthy body image (for example, a healthy diet, lifestyle, and physical activity) than on body size per se [48], and that it is necessary to balance weight management advice with supporting body satisfaction [10]. With regards to this, it is encouraging that this metasynthesis supports findings that pregnancy is a “teachable moment” [49] and that pregnant women are more receptive to conversations about weight-related aspects of their body image during pregnancy. Communication skills training may increase health professionals’ confidence in exploring women’s body image distress irrespective of the woman’s BMI, so they can be supported to either improve their relationship with their body image or engage in behaviours that would reconcile their desired and current body image; for example, through weight management strategies. There may also be usefulness in a broader programme of education aimed at the public about the implications of striving for the perfect body and the impact of the societal attitude on adjustment to pregnancy-related changes. A public health campaign may raise awareness of attitudes towards pregnancy that women perceive in the workplace, enabling women to feel the changes in their body are less likely to be in conflict with their working role.

Pregnant women undergo a psychological as well as physical adjustment process to a normal but altered state. This process has been described as developing a mothering orientation [50]. The significance of making the transition to motherhood should not be underestimated [51]. Women’s adjustment to the fetus and pregnancy-related physical changes may reflect their developing maternal orientation and the way in which they are adjusting to motherhood. Health professionals should note how women portray their fetus and bodily changes. Women who struggle to delineate between the body and the fetus, may view the fetus as an invasion or fail to accept some pregnancy changes, which could negatively impact on their ability to bond with their baby. Awareness of problematic attitudes may allow the opportunity to support women in reconciling their body image and bonding with first their fetus, then their baby.

## Conclusions

Pregnancy-related changes have a significant impact on women’s body image by transgressing the socially constructed ideal image. Women protect against this through delineating between ‘fatness’ and pregnancy, perceiving themselves excused. Striving to reclaim the postpartum body, women viewed the mothering identity conferred by their body as in conflict with other identities. Pregnancy presents an opportunity to provide healthy lifestyle advice focused on achieving a desired body image rather than a desired weight, in order to motivate women to consider weight-related behaviours in relation to their postpartum body, by offering advice or signposting to local services that will enable women to develop a healthy lifestyle. Women’s narratives about their fetus and their body may indicate those vulnerable to difficulties in adjusting to motherhood and subsequent bonding.
